# Hide-and-Seek in a Highly Human-Dominated Landscape: Insights into Movement Patterns and Selection of Resting Sites of Rehabilitated Wolves (*Canis lupus*) in Northern Italy

**DOI:** 10.3390/ani13010046

**Published:** 2022-12-22

**Authors:** Elisa Torretta, Andrea Corradini, Luca Pedrotti, Luciano Bani, Francesco Bisi, Olivia Dondina

**Affiliations:** 1 Department of Earth and Environmental Sciences, University of Pavia, Via Ferrata 1, 27100 Pavia, Italy; 2 Animal Ecology Unit, Research and Innovation Centre, Fondazione Edmund Mach, Via Edmund Mach, 1, 38098 San Michele all’Adige, Italy; 3 Stelvio National Park, 23032 Bormio, Italy; 4 Department of Earth and Environmental Sciences, University of Milano-Bicocca, Piazza della Scienza 1, 20126 Milan, Italy; 5 Environment Analysis and Management Unit, Guido Tosi Research Group, Department of Theoretical and Applied Sciences, Insubria University, Via J. H. Dunant, 3-I, 21100 Varese, Italy

**Keywords:** connectivity, dispersal, floaters, GPS telemetry, human disturbance, spatio-temporal segregation

## Abstract

**Simple Summary:**

In the last decades, a significant recovery and natural expansion of the wolf (*Canis lupus*) populations has occurred across Europe. This remarkable recolonisation was made possible by the high plasticity of the species, which was crucial in such a human-altered environment. Despite re-establishing within their former distribution range, understanding the behavioural responses adopted by this large carnivore to navigate in an increasingly anthropogenic world remains challenging. In this study, we investigated the movement ecology of three rehabilitated wolves in one of the most human-dominated landscapes of Europe, the Po Plain in Northern Italy, and obtained preliminary evidence of the ability of wolves to spatio-temporally segregate from human activities. We observed that (i) when wolves settled, they made considerably longer daily movements; (ii) when dispersing, wolves were more nocturnal in order to avoid encounters with humans; (iii) long-distance movements were aided by the availability of small-wooded patches used as resting areas. Our results provide important insight into the movement patterns of wolves in anthropogenic ecosystems, which may be used to inform future management actions that aim to facilitate wolf dispersal and settlement in human-dominated landscapes and to reduce human–wolf encounters, with the goal of promoting coexistence.

**Abstract:**

Assessing the behavioural responses of floating wolves to human presence is crucial for investigating the chance of wolf populations expanding into urbanised landscapes. We studied the movement ecology of three rehabilitated wolves in a highly human-dominated landscape (Po Plain, Italy) to explore wolf’s plasticity amid widespread human pressure. To reach this aim, we estimated individual 95% utilisation distributions (UD) after the release and inspected both 95% UDs and net squared displacements to identify individual movement patterns; tested for differences in movement patterns during day and night; and analysed the selection of resting sites during dispersal movement in a highly human-altered environment. Both the 95% UDs and step lengths were smaller for wolves settling in suitable areas than for those settling in more urbanised areas. All wolves exhibited strong temporal segregation with humans during all movement phases, particularly while dispersing across highly urbanised areas. Main roads and proximity to built-up areas were shown to limit wolves’ dispersal, whereas small-wooded patches that provide shelter during rest facilitated long-distance movements. This study provides important insights into wolf movement and settling in urban and peri-urban areas, providing critical knowledge to promote human–carnivore coexistence.

## 1. Introduction

In the last decades, a significant recovery of large carnivores has occurred across Europe; among them, the wolf (*Canis lupus*) is the most successful species in adapting to human-dominated landscapes with stable populations largely persisting outside protected and/or wilderness areas [1]. Wolves can persist in human-dominated landscapes by adjusting their behaviours to minimise the probability of direct encounters with humans [2,3,4]. To maximise spatio-temporal segregation from human activities, wolves can employ behavioural tactics, such as increased nocturnality [5,6,7], avoidance of areas with high human pressure [6,8,9,10,11,12], and selection of sites with dense vegetation cover for shelter [4,13]. This is true for both individuals living in territorial packs [4] and non-territorial wolves, i.e., floaters [14]; floaters are yearlings (12–23 months) or adults (≥24 months) that disperse from the natal pack to form their own pack or join an existing one [15], but also individuals that have become erratic as a result of pack dissolution due to natural or anthropogenic causes [16], or because forced to leave their original pack, e.g., old individuals [17]. Floaters move and generally settle in territories free from packs [17]; for this reason, they are usually the first to appear in the proximity of urban areas or in highly fragmented landscapes, such as agro-ecosystems or mosaics of woodland and farmland. Investigating the movement ecology of floaters is therefore essential to understand the spatial patterns and behaviours that allow wolf populations to eventually expand in human-modified landscapes [14,15,18].

Several aspects of wolf dispersal have been extensively investigated in North America [19,20,21,22,23,24] and Northern Europe [25,26,27,28,29], while limited information is available for Southern Europe [30,31,32], especially for highly urbanised and agricultural contexts [33], where conditions allow to investigate floaters’ movement ecology in human-modified landscapes [14]. For example, to our knowledge, there is only little information on the suitability of the few natural or semi-natural patches occurring in intensively modified agro-ecosystems to play the role of stepping-stones during wolves’ dispersal [34].

The dynamics of distribution and consistency of wolf population in Italy offer an interesting case study in this regard. After a severe decline that reduced the population of the Italian wolf (*Canis lupus italicus* Altobello, 1921) to less than a hundred individuals that survived in small, highly isolated areas of the Central and Southern Apennines [35,36], the population has rapidly increased starting in the early 1980s [37] and recolonized most of the Apennines over a couple of decades [38]. From the 1990s, dispersing individuals expanded from the Northern Apennines to the Western Alps through a mountainous ecological corridor, beginning the recolonisation of the Alps where they had been extirpated in the early 1900s [38]. A similar process occurred in the Eastern Alps, with dispersing individuals from the Dinaric–Balkan population (*Canis lupus lupus*) recolonizing the Alps westwards [39]. Currently, the Italian population is estimated to have a consistency of 3307 (range 2945–3608) wolves, of which 2388 (range 2020–2645) are in the Apennines and 946 (range 822–1099) are in the Alps [40].

**Figure 1:**
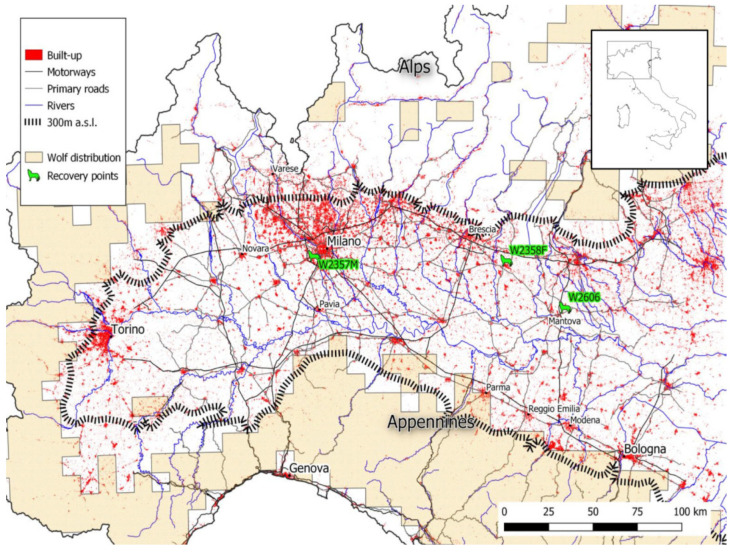
The study area in Northern Italy with wolf distribution, obtained from the report on the species of community interest (2013–2018) and updated with more recent data (2022), and recovery sites of the three wolves.

Within the Italian range, low population densities can be found in the lowlands and foothills bordering the Northern Apennines and the Central Alps [41], where scattered and recently established packs are present. Interestingly, areas recently recolonised by wolves generally show high dispersal rates [15]. This may be the reason why floaters have been increasingly sighted across the Po Plain (Figure 1), the highly human-modified flatland region located between the Eastern-Central Alps and the Apennines. These sightings have occurred not only in the few remaining natural protected areas, such as the Ticino River Natural Park [41,42], but also in the extremely simplified agro-ecosystems and large built-up areas.

In Italy, a few studies focused on the movement ecology of floating wolves and, despite individuals were live-trapped [14,43,44] or rescued after accidents [30,32], they were released in suitable areas characterised by low human density and high availability of refuge areas and wild prey. [14] argued that the behavioural plasticity (i.e., nocturnality and avoidance of human disturbance) observed for the radio-collared wolves in the Abruzzo Lazio and Molise National Park (PNALM; Central Apennines, Italy) was likely related to relatively suitable environmental conditions of the area of the release; the same responses may thus not necessarily be feasible or functional in human-dominated landscapes.

In this study, we investigated the movement ecology of three wolves rescued in areas far from stable pack territories, recovered, equipped with GPS collars, and hard-released (i.e., direct release without any previous acclimatisation) in three different areas within the Po Plain, one of the most highly human-dominated landscapes in Europe. The main objectives of the study are to provide insights regarding the movement behaviour of floaters in non-natural areas and to investigate whether wolves can adopt behavioural responses favouring the spatio-temporal segregation with human activities in highly human-modified landscapes. The specific objectives of the research were to investigate (i) individual utilisation distributions and movement patterns after hard-release; (ii) differences in movement patterns according to times of the day (day vs. night); and (iii) selection of resting sites during pre-dispersal and dispersal movements within the Po Plain.

## 2. Materials and Methods

### 2.1. Recovered Wolves

The three wolves were all rescued in Lombardy Region and moved to the wildlife rescue centre “C.R.A.S. Monte Adone” (Sasso Marconi, Italy) to assess their health condition and to receive the necessary medical treatments. The wolves remained in the facility for the shortest time possible (i.e., time strictly necessary for healing) and human contacts were reduced to the minimum necessary to avoid the emergence of stressful and stereotypical behaviours due to captivity. When they had fully recovered, i.e., when their physical condition allowed them to survive back in the wild, they were fitted with a GPS collar (Vectronic GPS–GSM collars, Vectronic Aerospace GmbH, Berlin, Germany) and hard-released in a natural or semi-natural location as close as possible to where they had been initially rescued. The release of the three wolves was approved by the Italian National Institute for Environmental Protection and Research (I.S.P.R.A.).

The first rescued and rehabilitated wolf (individual code: W2357M) was a 2-year-old male found in a canal in the suburbs of Milan on 23 April 2019. Because of a prolonged physical effort, it had an acute cardiorenal syndrome. Genetic analyses showed that the animal was an Italian wolf, with genetic traces of Slovenian and central European populations. It was released on 16 May 2019 in the Ticino River Natural Park, the nearest natural area to the rescue site. 

The second wolf (individual code: W2358F) was a 2-year-old female found in Lonato (Brescia Province) on the southwest shore of Lake Garda on 23 April 2019. The wolf had a severe urinary tract infection. Genetic analyses confirmed that the animal was an Italian wolf. It was released on 13 June 2019 near the recovery site.

The third wolf (individual code: W2606) was a 2-year-old female found in an agricultural area near Bigarello (Mantua Province) on 4 April 2021. Probably as a consequence of a vehicle collision, the wolf had a severe abdominal haemorrhage and femoral dislocation. Genetic analyses confirmed that the animal was an Italian wolf as well. It was released on 20 April 2021 near the recovery site. 

### 2.2. Study Area

The three wolves moved within a wide area in Northern Italy, across the Po Plain and the Northern Apennines, covering about 20,000 km^2^ (Figure 1). The Po Plain (<300 m a.s.l.) is a highly transformed landscape by agriculture and urbanisation (355 inhabitant/km^2^, twice the national average), with rice and corn being the most intensively cultivated crops. The region is also characterised by a vast and capillary infrastructure network, including roads and railways. Small and isolated wooded patches are interspersed within the landscape and mainly localised along rivers (Figure S1). The Northern Apennines (≥300 m a.s.l.), south of the Po Plain, is divided into a low hill area with extensive vineyards and a high hill/mountain area with continuous woodlands (detailed descriptions of the study area are available in [41,45,46]). 

### 2.3. Data Analyses

The GPS collars of the three wolves were programmed to acquire fixes every hour after the release and at different time intervals for the rest of the period depending on individual monitoring objectives. We considered different regular subsamples of GPS locations for each individual (W2357M: 6-h intervals (h: 00:00; 06:00; 12:00; 18:00); W2358F: 6-hour intervals (h: 04:00; 10:00; 16:00; 22:00); W2606: 4 h intervals (h: 00:00; 04:00; 08:00; 12:00; 16:00; 20:00)). The selected subsamples were the only ones that resulted in regular time intervals for each wolf, allowing for reliable estimates of utilisation distributions, movement patterns, and resting sites.

#### 2.3.1. Utilisation Distribution Estimation

We estimated the wolves’ utilisation distribution (UD) using a Brownian Bridge Movement Model (BBMM) [47] from the “BBMM” R package [48,49] and considering the area within the 95% isopleth as the main settlement area. This estimator lacks the assumption of independence among locations and explicitly incorporates the time lag and location error (set to 30 m, which is approximately twice the mean accuracy (8–15 m) claimed by the GPS collar provider, Vectronic Aerospace GmbH) between consecutive GPS locations to estimate the UD [47]. Importantly, taking into account time lag facilitated comparison of UD derived from trajectory with different time intervals. For each 95% UD (i.e., settlement area), we calculated the surface area (km^2^) and used them as a reference for classifying movement patterns outside the main settlement area.

#### 2.3.2. Movement Patterns

To identify movement patterns, we visually inspected the GPS trajectories in relation to the estimated 95% UD and net squared displacement (NSD) curve [50,51], which was plotted using the “adehabitatLT” R package [52,53]. Using these criteria, we distinguished five distinct movement patterns:

- Post-release: non-directional movements following the release and preceding other well-defined and recognisable movements (see below);
- Settlement: movements within a defined area (as defined by the 95% UD);
- Exploration: occasional movements beyond the 95% UD in never, or seldom, visited areas delineated by successive locations spanning from ≥12 h to <6 days [43,54];
- Pre-dispersal: directional movements beyond the 95% UD that preceded dispersal and lasted for more than 6 days, with wolves eventually returning to the release site or within the 95% UD [33,43];
- Dispersal: directional movements beyond the 95% UD [7], with wolves never returning to the release site or within the 95% UD.

To better inspect and describe the wolves’ movements patterns, we plotted the minimum daily distance and the minimum distance travelled, i.e., the cumulative line distance, as the sum of the Euclidean distances covered across all successive GPS locations. We also quantified the cumulative net displacement covered from the release date to the last day of monitoring [7,30] and the maximum net displacement as the Euclidean distance between the two farthest locations along the covered trajectory. We finally calculated the mean travel speed (km/h), as the mean of the distances covered across successive GPS locations divided by the time between successive locations [7].

Regarding the dispersal phase, we calculated the net dispersal distance, as the Euclidean distance between the release site and the last GPS location while dispersing [7] and between the release site and the farthest location along the dispersal trajectory [30,43].

#### 2.3.3. Differences in Step Lengths among Movement Patterns and Time of the Day

For each wolf, we classified the steps along the trajectory as both diurnal or nocturnal, excluding the steps occurring during dusk or dawn hours. The step length distribution is generally right skewed [55], and thus the Mann–Whitney test with permutation (*n* = 10,000) was used to test for differences in the distance between successive GPS locations (i.e., the steps) between day and night, and among the identified movement patterns, namely post-release vs. settlement vs. exploration vs. dispersal (i.e., pre-dispersal + dispersal). 

#### 2.3.4. Selection of Resting Sites along the Dispersal Trajectory within the Po Plain

Resting sites occurring during pre-dispersal and dispersal movements across the Po Plain were identified via spatio-temporal clustering of the wolf GPS locations. We identified hotspots of use (i.e., clusters) based on ad hoc parameterisation: a 250 m radius circle [4] was drawn around each GPS location, and the time spent inside that buffer was calculated using each GPS location’s timestamp. We categorised clusters as resting sites when they included locations for at least 6 consecutive hours [4]. The centroid based on all adjacent clusters was calculated, and that location was taken as the resting location when multiple buffers overlapped (within 500 m).

We tested the selection of resting sites below 300 m a.s.l. (i.e., the Po Plain) and along the pre-dispersal and dispersal trajectories as a discrete choice influenced by movement, using a matched case-control approach, where each resting location (case, or used) is matched with a conditional availability set (control, or available). For each resting site (t), we identified the previous GPS location (t–t_n_) and generated 25 random locations using the empirical distribution of each wolf’s step length and turning angle. We extracted for each set of locations (used and available) ecologically meaningful environmental covariates to evaluate wolves’ selection of resting sites within the landscape. We tested the individual selection of environmental conditions by applying a conditional logistic regression (CLR) using the Cox proportional-hazards model from the R packages “survival” [56,57]. We included land cover information at 10 m spatial resolution [58]. We thus defined tree and human settlement density as the mean tree (European Union, EEA 2018) and human settlement density [59], respectively, in a circular buffer for each site with different radii (50, 100, 250, and 500 m). Minimum distance from roads (map data copyrighted by OpenStreetMap contributors and available from https://www.openstreetmap.org; accessed on 5 September 2022) and rivers (Ministero Ambiente; http://www.pcn.minambiente.it/mattm/servizio-wms/; accessed on 5 September 2022) was calculated with the “dist2Line” function of the R package “geosphere” [60]. Distances from tree and built patches were calculated on the land cover grid with the “distance” function of the R package “raster” [61]. To reduce the impact of linear features over large distances, all distance covariates were converted to exponential decays, with values ranging from 0 (at distance = 0) to 1 (for large distance values) (modified from [62]). Finally, based on available land cover information [58], we computed landscape metrics at different buffers (250 and 500 m radii) using the “landscapemetrics” R package [63] (Table S1). We selected the most meaningful radius of influence for each density covariates and landscape metrics through a preliminary model selection (Table S2), as well as a correlation matrix analysis to select the best uncorrelated covariates to include as predictors in the individual models (Figure S2). To assess the relative importance of each covariate, we performed model selection via ‘dredging’, that is the repeated evaluation of the set of models with all possible combinations (until the ‘null model’) of fixed effect terms in the full model, using the “dredge” function of the R package “MuMIn” [64]. We derived model-averaged coefficients (full average) by considering all models with the lowest Akaike information criterion (AIC) score (≤2, with respect to the best model) and evaluated the contribution of each covariate, particularly those that were statistically significant [65].

Finally, the temporal distribution of resting locations according to the time of day was also investigated. We determined the level of diurnality for each site as the percentage of time the wolf rested during the day compared to the night (from 0, fully nocturnal, to 1, fully diurnal) and looked for statistically significant variations in the level of diurnality among individuals by using a pairwise Wilcoxon test. 

We cannot exclude that the pre-release events (i.e., accident, rescue, and recovery) might have influenced wolves’ behaviour; for example, the habituation to human presence might have caused confident behaviours particularly evident in urban and peri-urban areas, but such effects remain largely unknown.

## 3. Results

### 3.1. Utilisation Distribution and Movement Patterns

**Figure 2:**
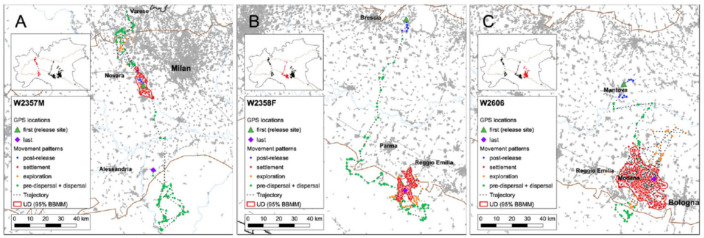
GPS locations obtained for three GPS-collared wolves in Northern Italy, 2019–2021. (**A**) W2357M; (**B**) W2358F; (**C**) W2606. Brown line delineates 300 m a.s.l. contour line, while urban areas are in grey.

**Figure 3:**
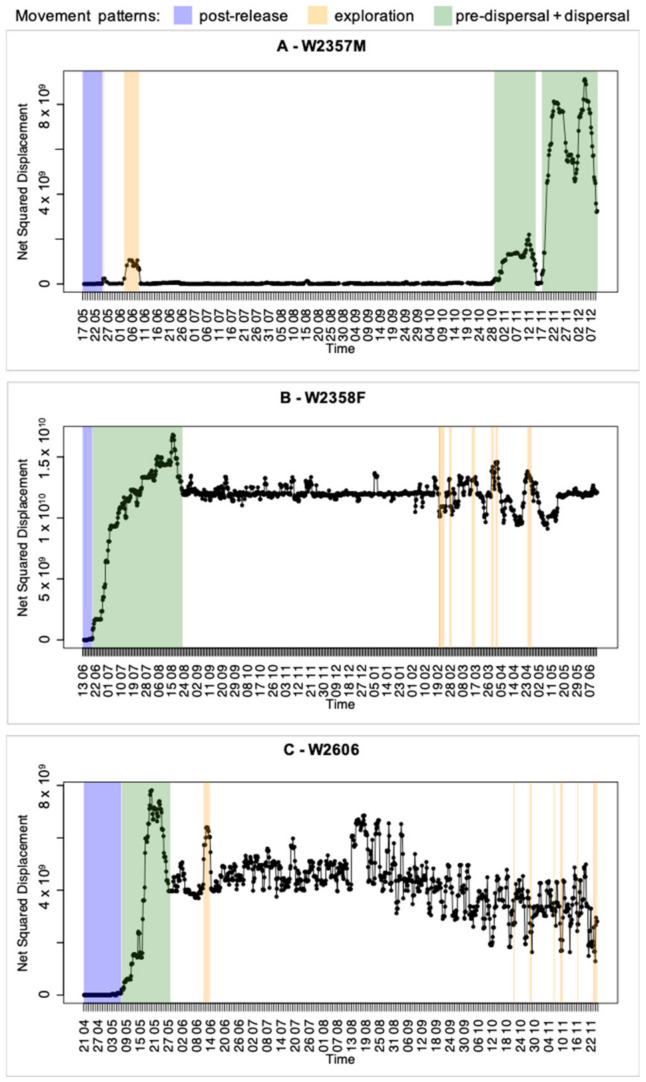
Net squared displacement and identified movement patterns of three GPS-collared wolves in Northern Italy, 2019–2021. (**A**) W2357M; (**B**) W2358F; (**C**) W2606.

The collar of the wolf W2357M worked correctly for 207 days, from 16 May to 10 December 2019; during this period, we obtained 771 GPS locations useful for the data analyses. The 95% UD of the wolf measured 98.2 km^2^ and it was located around the release site, within the Ticino River Natural Park (Figure 2A). After the post-release period, the wolf made two exploration movements northwards, along the Ticino River, followed by a prolonged settlement phase, then a pre-dispersal movement occurred (~50 km northwards), and, finally, a dispersal movement southward to the Ligurian Apennines (Figure 2A and Figure 3A; Table S3). The wolf covered a cumulative net displacement of 55.1 km and a cumulative line distance of 1182.8 km (Figure S3A and Table 1). The net dispersal distance was 55.1 km, considering the last GPS location while dispersing, or 94.0 km, considering the farthest location along the dispersal trajectory (Figure 2A and Table 1). 

The collar of W2358F worked correctly for 365 days, from 13 June 2019 to 12 June 2020; during this period, we obtained 1437 GPS locations useful for the data analyses. The 95% UD of the wolf measured 114.2 km^2^ (Figure 2B). After the post-release period, the wolf immediately dispersed travelling southwards moving away for approximately 120 km from the release site. The wolf settled in a hilly area on the border of the Apennines of Reggio Emilia (Emilia–Romagna Region). After a prolonged stationary period, the wolf started the exploration phase, during which we observed seven different exploration events (Figure 2B and Figure 3B; Table S3). The wolf covered a cumulative net displacement of 110.0 km and a cumulative line distance of 1649.2 km (Figure S3B and Table 1). The net dispersal distance was 109.4 km, considering the last GPS location while dispersing, or 129.7 km, considering the farthest location along the dispersal trajectory (Figure 2B and Table 1). 

The collar of W2606 worked correctly for 218 days, from 21 April to 25 November 2021; during this period, we obtained 1310 GPS locations useful for the data analyses. The 95% UD of the wolf measured 638.2 km^2^ (Figure 2C). After the post-release period, the wolf immediately dispersed travelling southwards moving away for approximately 70 km from the release site. The wolf settled in an urbanised area around the city of Modena. We observed a first exploration event a little while later the settlement and, similarly to W2358F, after a prolonged stationary period, the wolf started the exploration phase, during which we observed other six exploration events (Figure 2C and Figure 3C; Table S3). The wolf covered a cumulative net displacement of 70.1 km and a cumulative line distance of 2922.4 km (Figure S3C and Table 1). The net dispersal distance was 62.9 km, considering the last GPS location while dispersing, or 88.4 km, considering the farthest location along the dispersal trajectory (Figure 2C and Table 1).

### 3.2. Differences in Step Lengths among Movement Patterns and Time of the Day

The nocturnal steps of W2357M and W2606 were significantly longer than the diurnal ones for each movement pattern, while the nocturnal steps of W2358F were significantly longer than the diurnal ones for each movement pattern with the exception of post-release movements (Table 2). 

**Figure 4:**
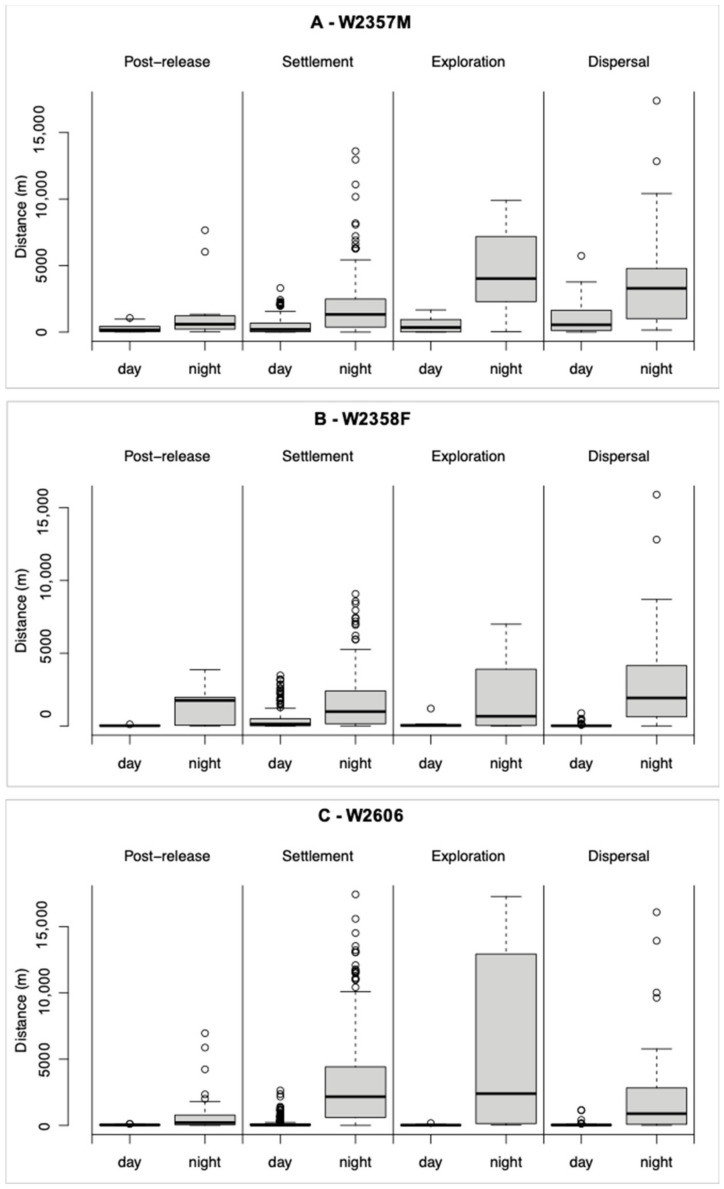
Diurnal and nocturnal step lengths for each movement pattern of three GPS-collared wolves in Northern Italy, 2019–2021. (**A**) W2357M; (**B**) W2358F; (**C**) W2606.

Considering the diurnal steps of W2357M, significant differences only emerged comparing settlement vs. dispersal patterns; conversely, the nocturnal steps greatly differed between patterns, with the exception of release vs. settlement patterns and exploration vs. dispersal patterns (Table 3; Figure 4A). Considering the diurnal steps of W2358F, significant differences emerged comparing settlement vs. dispersal and settlement vs. exploration patterns; similarly, also considering the nocturnal steps, significant differences emerged comparing settlement vs. dispersal (Table 3; Figure 4B). Finally, considering the diurnal steps of W2606, significant differences only emerged comparing settlement vs. exploration patterns; considering the nocturnal steps, significant differences emerged comparing settlement vs. dispersal, release vs. exploration, and release vs. settlement (Table 3; Figure 4C). 

### 3.3. Selection of Resting Sites along the Dispersal Trajectory

**Figure 5:**
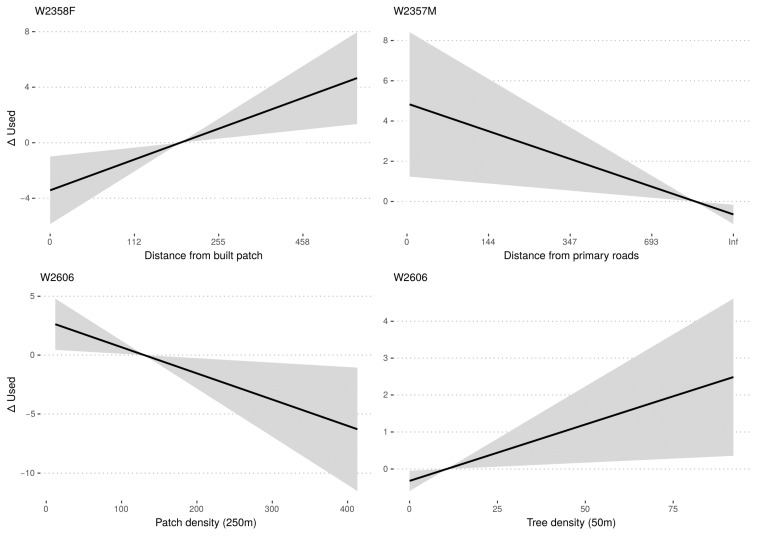
The fitted regression lines with standard error showing the statistically significant environmental predictor that drives individual selection of resting sites, as estimated by conditional logistic regression.

For W2357M, we identified a total of 26 resting sites, 13 of which during pre-dispersal and dispersal phases, and observed that resting sites below 300 m a.s.l. (Po Plain) were significantly closer to primary roads than random, while we found no significant relationship with any other covariate (Table 4, Figure 5). Model selection (i.e., dredge) corroborated this result, which also highlighted the relative importance of distance from motorways, tree density (50 m), human settlement density (250 m), and patch density (250 m), despite these predictors not being significant as single predictors (Table S4). 

For W2358F, we identified a total of 46 resting sites, but only 11 of these occurred during dispersal movements below 300 m a.s.l. W2358F selected resting sites in areas significantly further away from built patches, and importantly was the only wolf to show this behaviour, with no other environmental variables showing to be significantly important (Table 4, Figure 5). Model selection (i.e., dredge) supported this finding as well, and showed the relative importance of patch density (250 m) and distance from rivers, despite these predictors not being significant as single predictors (Table S4). 

Finally, for W2606, we identified a total of 21 resting sites, 16 of which were along the dispersal path in the highly disturbed Po Plain. Resting sites were found in locations with significantly higher tree density (50 m) and lower patch density (250 m) than random (Table 4, Figure 5). The selection of the most significant predictors was further reinforced by model selection (i.e., “full” model-averaged coefficients), which also indicated the relative importance of the distance from highways and rivers (Table S4).

**Figure 6:**
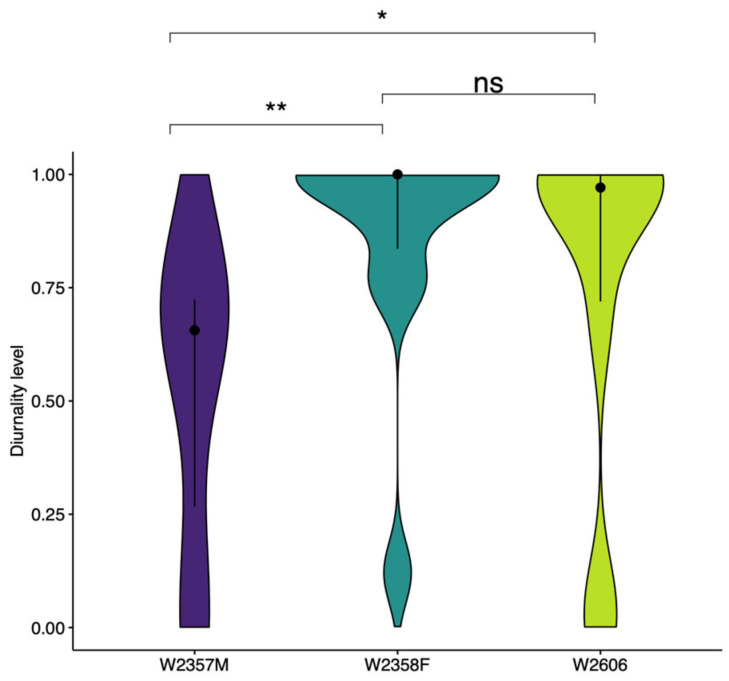
Violin plots with the distribution of resting sites based on their level of diurnality (from 0, fully nocturnal, to 1, fully diurnal), for each wolf. The median (Q2, black dot) and the interquartile range (Q1–Q3, solid line) are shown for reference. Pairwise Wilcoxon test comparisons between individuals, with associated significance levels, are reported above each plot. ns. not significant; * *p* < 0.05; ** *p* < 0.01.

W2357M rested primarily during the daytime and twilight hours and its resting behaviour differed significantly from that of the two female wolves (W2358F and W2606), which selected resting sites almost exclusively during the day (Figure 6).

## 4. Discussion

In the plethora of studies that focused on wolf behavioural responses to human disturbance [4,6,8,13,66,67], our research provided rare evidence of the use of space and the movement behaviour of wolves within one of the most human-dominated landscapes of Europe. As mentioned before, our data were collected from rescued wolves, we thus have no information on the movements of the three wolves before their recovery and we cannot exclude that the pre-release events (accident, rescue, and recovery) might have influenced their behaviour. Nevertheless, given the limited handling time and contact with humans during the recovery of all three wolves, we are confident that conditioning or habituation effects did not occur, or were negligible [30].

### 4.1. Utilisation Distribution and Movement Patterns

The extent of the area occupied by W2357M and W2358F was included within the range estimated for resident pack members in Central Europe (MCP (Minimum Convex Polygon) 100%: 82−243 km^2^) [68] and Southern Europe; in Italy, for example, both the home range of a floating wolf when it settled on the Maritime Alps, Italy, (95% fixed kernel: 71.8 km^2^) [30] and the mean annual home range of resident pack members (BBMM 95%: 104 km^2^) in Abruzzo, Lazio and Molise National Park (PNALM), Italy, [14] were similar. Conversely, the area where W2606 settled was significantly larger than the area occupied by the other two wolves in this study, but also by floaters in the PNALM (BBMM 95%: 293.8–408.7 km^2^) [14]. It has been demonstrated that wolves’ home ranges are negatively related to prey abundance and habitat quality [21,69] and positively related to human density [70] and habitat fragmentation caused by roads [14]; these two factors generally force wolves to increase their range size to encompass refuge areas large enough to satisfy their ecological needs. W2606 settled in the suburban area of Modena, a highly urbanised area extremely fragmented by roads and lacking stable populations of wild ungulates [71], while W2357M and W2358F settled in more natural areas characterised by continuous woodlands and high prey availability (i.e., the Ticino River Natural Park: 30.7 ± 4.1 roe deer/km^2^ [72]; Apennines, hilly areas between Parma and Reggio-Emilia: stable occurrence of roe deer and wild boar [71]). These differences in refuge and prey availability could have driven the observed differences in the utilisation distribution of the three monitored wolves.

The larger minimum daily distance travelled by W2606 with respect to both W2357M and W2358F and the larger step lengths at night during the settlement phase further support the hypothesis that W2606 needed to cover larger distances to find shelter and food resources in a degraded landscape. Even if the minimum daily distance could have been partially influenced by the higher relocation frequency of W2606 compared to that of the other two wolves (4 h for W2606 versus 6 h for W2357M and W2358F) [15], the observed mean travel speed, which is a measure independent from relocation frequencies, was higher as well, supporting this hypothesis.

The settlement area of W2357M was located around the release site and was occupied by the wolf for most of the monitoring period; only by the end of this period did the wolf begin a dispersal movement. In contrast, both W2358F and W2606 dispersed shortly after the release and then settled in an area located further away (>50 km) from the release site. The net dispersal distance observed for the three wolves, which essentially resulted in the observed cumulative net displacement, was comparable to the dispersal distances observed for wolves in Southern Europe ([30,32,33,43,73,74,75]; but see [31]), while it was noticeably lower than dispersal distances observed in Northern Europe [15], Asia [76,77], and North America [15]. We can hypothesize that the lower values of dispersal distances observed for the monitored wolves could be attributed to the high degree of habitat fragmentation and urbanisation of the areas they crossed; highly modified landscapes are known to shorten dispersal distances due to their low permeability to wolves’ movements [33]. Interestingly, all wolves dispersed in roughly the same direction, that is southward, although, for example, in the case of W2358F, the nearest natural areas were northwards. Observations of similar dispersal behaviour are increasing [7,78,79]. One hypothesis is that these parallel movements were driven by landscape features that facilitate movement [79] or, as an alternative hypothesis, that wolves moved towards their natal areas. However, it remains an unexplained dispersal pattern, and further research is needed to shed light on possible drivers [7]. 

### 4.2. Differences in Step Lengths among Movement Patterns and Time of the Day

We distinguished five patterns of movement along the wolves’ trajectories: post-release, settlement, exploration, pre-dispersal, and dispersal. During each phase, all wolves made significantly longer movements at night than during the day, except for the post-release phase of W2358F, likely because of the low sample size (*N* = 6). This result suggests that wolves could have temporally segregated from human activities [5,6,7] while crossing, and resting, in areas intensively used by humans.

During the first 6–15 days after the release (i.e., post-release phase), all the three wolves remained in a very small area and travelled very short distances compared to other movement phases. In Italy, a comparable pattern was reported by [30] and [32] for rehabilitated wolves that remained within a circumscribed area for ten days and two months, respectively, after their release. We hypothesize that this behaviour could be related to the unfamiliarity with the release area that likely increased their risk perception to potential threats.

Immediately after the post-release phase, both W2358F and W2606 dispersed unidirectionally southward. Unidirectional dispersals generally underline the urgency of wolves to get quickly to a new area; travelling far along a straight line will ensure that the environmental conditions at the arrival will be sufficiently different from those at the starting point [79]. This behaviour could suggest that the environmental conditions at/near the release site of both W2358F and W2606 were highly unsuitable for their settlement and forced them to move rapidly away in search of a more suitable area. Both W2358F and W2606 settled after an evident backtrack in their trajectory, suggesting that they identified a suitable area along their dispersal trajectory for settlement [12], likely based on both environmental conditions and an absence of stable wolf packs. After settling in, both W2358F and W2606 did not leave the settlement area for about six and four months, respectively. Subsequently, they both started exploration movements, possibly in search of additional food sources [43,80,81,82] or a mate.

On the other hand, W2357M, except for two brief exploration events after the post-release phase, settled for about five months close to the release site. This behaviour could suggest that the conditions the wolf found at the release site were probably suitable to satisfy its primary ecological needs. Unlike W2358F and W2606, both of which were released within the intensively cultivated area of the Po Plain, W2357M was released within a natural protected area characterised by the presence of continuous woodlands and a rich and diverse wild ungulate community [42], which provided refuge and food resources over the long-term. The wolf finally left the settlement area and dispersed southward. Some evidence, such as the period when dispersal occurred (i.e., late autumn) and the typical pre-dispersal behaviour [7,20,79,83,84] could suggest that the dispersal of W2357M was not forced by the need to move away from an unsuitable area, but by the need to find a mate and establish a new pack, i.e., the main reason for wolves’ dispersal [17,85]. Nonetheless, we cannot rule out the possibility that this pre-dispersal event was a failed attempt to disperse northward. In fact, the northernmost GPS location recorded during this movement occurred along a hypothetical corridor (the Ticino River Natural Park), but in an area where an abrupt decline of ecological connectivity was predicted due to human settlements and roads [41]; moreover, during the last few years, two wolves were killed by car accidents exactly in this area. After the unidirectional dispersal southward, the NSD of W2357M showed two peaks and a turnaround, suggesting it had not found an available area to settle in the Northern Apennines, probably due to high pack density characterising the area [45,86,87].

Differences in the magnitude of movements travelled by wolves during the day and during the night also could reflect differences of habitat suitability in the areas where wolves settled and moved. In daylight hours, W2357M showed a behaviour typically observed in wolves [12], with a greater mean step length during movements outside the settlement area, especially during pre-dispersal and dispersal, than within it. Conversely, both W2358F and W2606 showed greater mean step lengths during movements within the settlement area than during exploration and dispersal. This could be due to the high unsuitability of the landscape crossed by these two wolves during movements outside the settlement area, which forced them to stay or make minimal movements during daylight hours, when human disturbance is high [3]. During the night hours, however, all three wolves showed greatest step lengths when moving outside the settlement area, suggesting that temporal segregation from human activities allows wolves to occupy highly human-dominated areas. 

### 4.3. Selection of Resting Sites along the Dispersal Trajectory

The temporal segregation from human activities during the pre-dispersal and dispersal periods also emerged from the significant differences in diurnality levels observed among resting sites. W2358F and W2606 dispersed through an area heavily used by humans and temporally segregated by resting during the day and moving during the night. The same behaviour was observed by [30] for a wolf that travelled faster during the night and rested during the day when it crossed the most cultivated and developed area along its trajectory. In contrast, W2357M travelled through a more natural environment and showed a lower diurnality while selecting for resting sites, which is consistent with findings from [3,88], who observed that the temporal avoidance of human activity rose as the degree of anthropogenic disturbance in the landscape increased.

The variables that most influenced the selection of resting sites also suggested a possible effect of the difference in the suitability of the areas crossed by wolves during pre-dispersal and dispersal on their dispersal behaviour. W2357M pre-dispersal and dispersal movements were substantially disturbed by the presence of main roads. Although highways and roads may reduce the rate of movement [89] and landscape connectivity for wolves [41,90], there is general evidence that individuals can overcome infrastructures [15,91], yet heavily trafficked roads remain difficult to overcome, especially for floaters without prior spatial knowledge of the area [30]. Ref. [30] observed that a dispersing wolf rested for four days close to a motorway before crossing it. We could infer that W2357M exhibited a similar behaviour because it could not cross these obstacles during daytime, thus resting in their proximity and waiting for night hours when vehicular traffic was lower.

In contrast, as noted above, the dispersal movements of W2358F and W2606 were interrupted by the need to seek for shelters during daylight hours, despite exhibited by different selection patterns: W2358F preferred resting far from built-up areas, while W2606 rested in areas with high, and homogenous, tree cover density. There are two possible explanations for the observed difference: (i) the territory crossed by W2606 had no sites sufficiently distant from built-up areas to be selected because of the high urbanisation [3], (ii) the two wolves had different level of tolerance towards human proximity: W2606 could sustain higher level of human presence than W2358F as far as there were shelter areas (i.e., small-wooded patches), similar to what was observed by [30] in a highly modified agriculture landscape in Northwestern Spain. Beyond the underlying cause, during dispersal, W2606 encountered highly modified conditions and, according to [12], this could have made it more tolerant toward human disturbance, eventually leading it to settle in a peri-urban area. 

## 5. Conclusions

Assessing wolf behavioural responses in human-dominated landscapes is critical to guide wolf conservation and to better understand the human–predator conflict. This study yielded several preliminary pieces of evidence regarding the ability of rehabilitated wolves to implement adaptive behavioural responses in highly urbanised areas, which can be summarised as follows:

- When wolves settle in urban or peri-urban areas, they make considerably longer movements, both within and outside the settlement area, probably to compensate for the paucity of food resources, especially wild prey, typical in highly human-dominated areas. This increases the chance of risky situations for the predator and increases the frequency of encounters with humans, thus the human perception of conflict [92,93].
- Environmental conditions at the release site could influence the movement patterns of rescued wolves during the subsequent phases.
- Landscape fragmentation caused by main roads can greatly slow down wolf dispersal.
- In areas with high human presence, wolves temporally segregate from human activities while dispersing.
- In areas with high urbanisation levels, small woodland patches could provide temporary shelter, allowing wolves to traverse highly disturbed areas by resting during the day and moving at night. Small wooded patches raise the ecological connectivity for wolves and other species [94] and reduce the encounter rate between wolves and humans, promoting their coexistence [4].

## Figures and Tables

**Table 1:** Covered distances and mean travel speed (km/h) of three GPS-collared wolves in Northern Italy, 2019–2021.

	A—W2357M	B—W2358F	C—W2606
Minimum daily distance (mean ± SD) (km)	5.4 ± 5.2	4.5 ± 3.2	13.4 ± 8.6
Cumulative net displacement (km)	55.1	110.0	70.1
Maximum net displacement (km)	142.3	129.8	91.3
Cumulative line distance (km)	1182.8	1649.2	2922.4
Mean travel speed (mean ± SD) (km/h)	0.24 ± 0.35	0.19 ± 0.26	0.57 ± 0.83
Dispersal [release—last GPS location](km)	55.1	109.4	62.9
Dispersal [release—farthest GPS location] (km)	94.0	129.7	88.4

**Table 2:** Differences in diurnal and nocturnal step lengths (m) (mean ± SD) within each movement pattern of three GPS-collared wolves in Northern Italy, 2019–2021. U = Mann–Whitney test.

Movement Pattern	A—W2357M		B—W2358F		C—W2606	
	Day	Night	Day	Night	Day	Night
Post-release	317.3 ± 352.8	1413.9 ± 2273.2	34.1 ± 41.7	1564.8 ± 1433.6	42.7 ± 29.0	953.8 ± 1694.0
	U = 0.744; *p*-value = 0.028		U = 0.778; *p*-value = 0.129		U = 0.795; *p*-value < 0.0001	
Settlement	432.3 ± 562.2	1816.0 ± 2034.0	420.4 ± 668.6	1569.5 ± 1776.8	137.6 ± 319.3	3144.1 ± 3280.9
	U = 0.797; *p*-value < 0.0001		U = 0.729; *p*-value < 0.0001		U = 0.912; *p*-value < 0.0001	
Exploration	536.9 ± 623.8	4656.0 ± 3149.7	120.7 ± 301.3	2096.5 ± 2472.8	31.1 ± 39.9	5852.0 ± 6674.3
	U = 0.912; *p*-value = 0.002		U = 0.728; *p*-value = 0.036		U = 0.920; *p*-value < 0.0001	
Dispersal	1045.4 ± 1167.7	3699.0 ± 3330.4	58.5 ± 140.8	2890.6 ± 3122.0	101.4 ± 246.5	2327.1 ± 3721.2
	U = 0.807; *p*-value < 0.0001		U = 0.950; *p*-value < 0.0001		U = 0.823; *p*-value < 0.0001	

**Table 3:** Differences in diurnal and nocturnal step lengths between different movement patterns of three GPS-collared wolves in Northern Italy, 2019–2021. U = Mann–Whitney test.

Movement Pattern	A—W2357M				B—W2358F				C—W2606			
	Day		Night		Day		Night		Day		Night	
	U	*p*- Value	U	*p*- Value	U	*p*- Value	U	*p*- Value	U	*p*- Value	U	*p*- Value
Post-release vs. Settlement	0.503	0.976	0.637	0.074	0.731	0.052	0.508	0.951	0.513	0.817	0.760	< 0.001
Post-release vs. Exploration	0.519	0.912	0.793	0.014	0.578	0.631	0.523	0.903	0.688	0.028	0.728	0.009
Post-release vs. Dispersal	0.671	0.050	0.777	<0.001	0.521	0.879	0.624	0.326	0.536	0.620	0.624	0.067
Settlement vs. Exploration	0.527	0.798	0.782	0.002	0.664	0.031	0.501	0.970	0.644	0.035	0.553	0.465
Settlement vs. Dispersal	0.661	<0.001	0.704	<0.001	0.723	< 0.001	0.642	<0.001	0.503	0.961	0.632	0.004
Exploration vs. Dispersal	0.625	0.251	0.597	0.322	0.607	0.207	0.621	0.161	0.650	0.063	0.640	0.097

**Table 4:** Output of the fitted individual-based models. For each individual model, the explanatory variables, parameter estimates (and 90% confidence interval), and significance levels (in bold when significant) are reported. The number of resting sites used to fit each model is reported below. * *p* < 0.05; ** *p* < 0.01.

	Coefficients (90% CI)		
	A—W2357M	B—W2358F	C—W2606
Tree density (50 m)	0.956 (0.045, 1.866)	0.143 (−0.795, 1.081)	0.962 * **(0.271, 1.653)**
Human settlement density (250 m)	−1.838 (−4.290, 0.613)	−1.264 (−4.870, 2.341)	−0.271 (−1.875, 1.333)
Patch density (250 m)	−0.384 (−1.662, 0.894)	1.388 (−0.084, 2.860)	−1.572 * **(−2.668, −0.476)**
Distance from motorways	−0.357 (−0.660, −0.054)	−0.348 (−1.650, 0.954)	19.642 (−50.430, 89.715)
Distance from primary roads	−0.839 ** **(−1.363, −0.315)**	0.069 (−1.251, 1.389)	−0.248 (−0.800, 0.304)
Distance from rivers	−0.371 (−0.855, 0.112)	−0.322 (−0.966, 0.323)	−0.353 (−0.772, 0.065)
Distance from built patch	0.115 (−0.710, 0.939)	2.234 ** **(0.902, 3.567)**	0.140 (−0.507, 0.788)
Observations	13	11	16

## Data Availability

Data are available on reasonable request from the corresponding author.

## Supplementary Materials

The following supporting information can be downloaded at: https://www.mdpi.com/article/10.3390/ani13010046/s1, Figure S1: The study area; Table S1: Landscape metrics included in model analysis; Table S2: Meaningful radius of influence of covariates; Figure S2: Correlation matrix of covariates; Table S3: Movement patterns; Figure S3: Minimum daily distance and cumulative line distance; Table S4: Relative importance of covariates obtained through multi-model inference.
