# Supplementary material for: Hide-and-Seek in a Highly Human-Dominated Landscape: Insights into Movement Patterns and Selection of Resting Sites of Rehabilitated Wolves (Canis lupus) in Northern Italy
Source: Animals (Basel). 2022 Dec 22;13(1):46. doi: 10.3390/ani13010046 (PMC9817923; doi:10.3390/ani13010046)
Supplement: Supplementary file 1 [file animals-13-00046-s001.zip › animals-2050242-supplementary.pdf]

SUPPLEMENTARY MATERIALS

Hide-and-Seek in a Highly Human-Dominated Landscape: Insights into Movement Patterns and Selection of Resting Sites of Rehabilitated Wolves (*Canis lupus*) in Northern Italy

Elisa Torretta, Andrea Corradini, Luca Pedrotti, Luciano Bani, Francesco Bisi[ǂ] and Olivia Dondina[ǂ*]

[ǂ] These authors contributed equally to this work.

[*] Correspondence: olivia.dondina@unimib.it

Figure S1: The study area.

A land cover map of the study area.

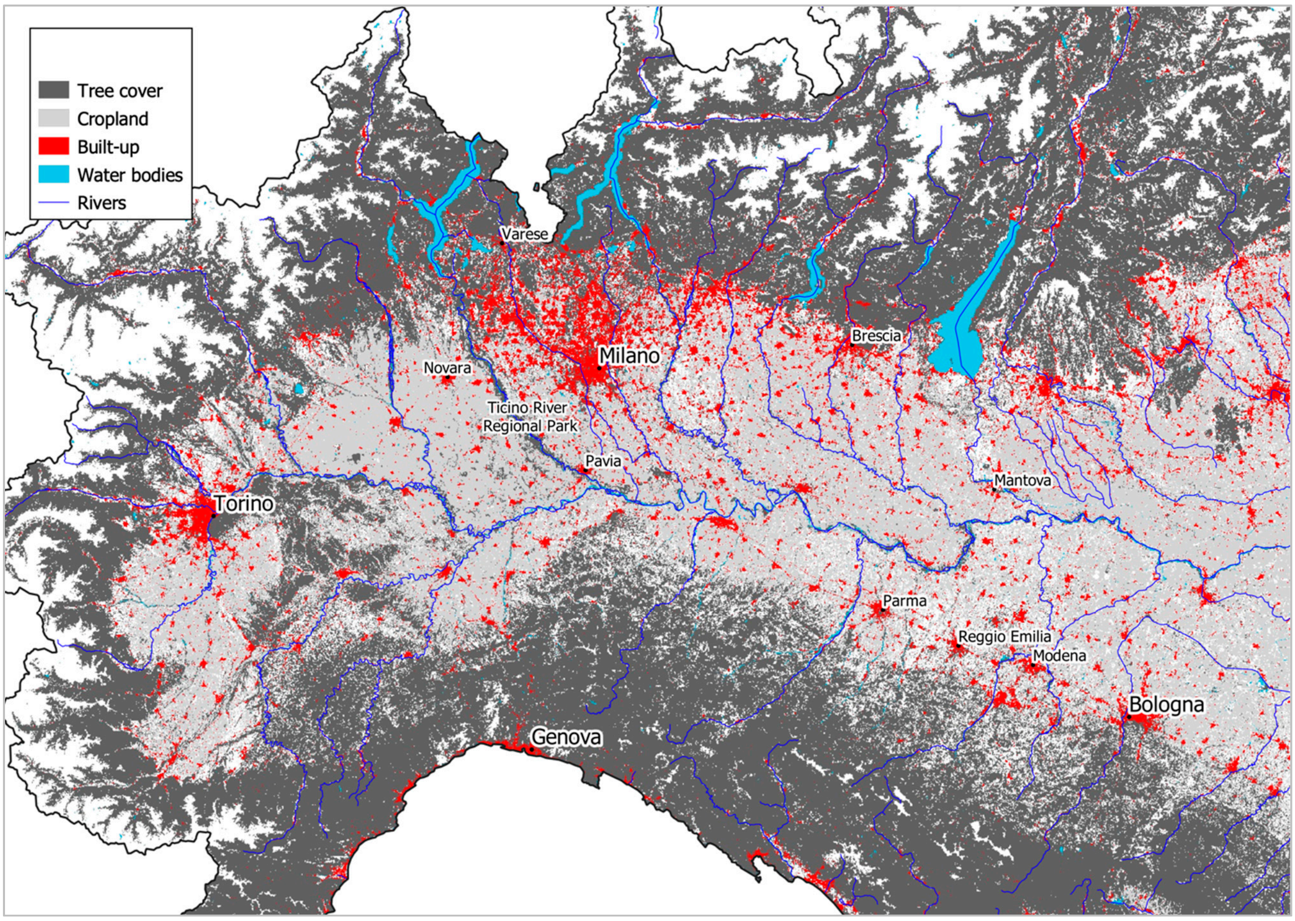

Table S1: Landscape metrics included in model analysis.

All the metrics have been calculated within two circular buffers around resting locations: 250 m and 500 m.

Name	Description
**ai**	Aggregation index: equals 0 for maximally disaggregated and 100 for maximally aggregated classes
**area mn**	Mean of patch area: equals 0 if all patches are small; increases (without limit) as the patch areas increase
**np**	Number of patches: equals 1 when only one patch is present and increases (without limit) as the number of patches increases
**pd**	Patch density: increases as the landscape gets more patchy. Reaches its maximum if every cell is a different patch
**pr**	Patch richness: equals 1 when only one patch is present and increases (without limit) as the number of classes increases
**prd**	Patch richness density: equals 1 when only one patch is present and the landscape is rather large; increases (without limit) as the number of classes increases and the total landscape area decreases

Table S2: Meaningful radius of influence of covariates.

For each wolf, the set of univariate models was fitted to determine, via model selection, the most meaningful radius of influence for each covariate (i.e., tree cover density, human settlement density, and landscape metric). In bold all the best performing univariate models (ΔAIC ≤ 2). The best performing radius across individuals, used in the formulation of the full model, is underlined.

	**AIC (ΔAIC from the best model)** significance level		
	**A - W2357M**	**B - W2358F**	**C - W2606**
TREE COVER DENSITY			
50m-radius buffer	**160.0 (0.8) ****	**292.3 (0) ****	**128.4 (0) ****
100m-radius buffer	**159.2 (0) ****	**292.7 (0.4) ****	**130.4 (2) ***
250m-radius buffer	162.6 (3.4)	296.1 (3.8) *	130.7 (2.3) *
500m-radius buffer	166.6 (7.4)	300.2 (7.9)	135.8 (7.4)
HUMAN SETTLEMENT DENSITY			
50m-radius buffer	167.0 (6.9)	285.5 (5)	136.2 (8.7)
100m-radius buffer	**160.7 (0.6)**	**280.5 (0) ***	133.3 (5.8)
250m-radius buffer	**160.1 (0) ***	**281.8 (1.3) ****	**127.5 (0) ***
500m-radius buffer	**162.0 (1.9) ***	289.7 (9.2) **	137.2 (9.7)
LANDSCAPE METRICS			
Aggregation index (250m)	**159.4 (0.3) ****	300.6 (2.8)	**123.3 (1.7) *****
Aggregation index (500m)	164.7 (5.6) *	301.4 (3.6)	131.4 (9.7) **
Mean of patch area (250m)	171.3 (12.2)	301.5 (3.7)	137.4 (15.7)
Mean of patch area (500m)	170.9 (11.8)	301.6 (3.8)	137.3 (15.6)
Number of patches (250m)	**159.1 (0) ****	**298.7 (0.9)**	**121.7 (0) *****
Number of patches (500m)	165.2 (6.1) *	**298.6 (0.8)**	129.6 (7.9) **
Patch density (250m)	**159.1 (0) ****	**298.7 (0.9)**	**121.8 (0.1) *****
Patch density (500m)	165.2 (6.1) *	**298.7 (0.9)**	129.6 (8) **
Patch richness (250m)	170.8 (11.7)	**297.9 (0.1) ***	133.0 (11.3) *
Patch richness (500m)	171.4 (12.3)	301.7 (3.9)	138.7 (17)
Patch richness density (250m)	170.8 (11.7)	**297.8 (0) ***	133.2 (11.5) *
Patch richness density (500m)	171.4 (12.3)	301.7 (3.9)	138.7 (17)
Note:	*p<0.05; **p<0.01; ***p<0.001		

Figure S2: Correlation matrix of covariates.

Correlation matrix with the Pearson correlation coefficients between each covariate computed for the formulation of models to test the individual selection of resting sites. For tree cover (td) density, human settlement (HS) density, and each landscape metric, only the best-performing buffer radius was selected (Table S2).

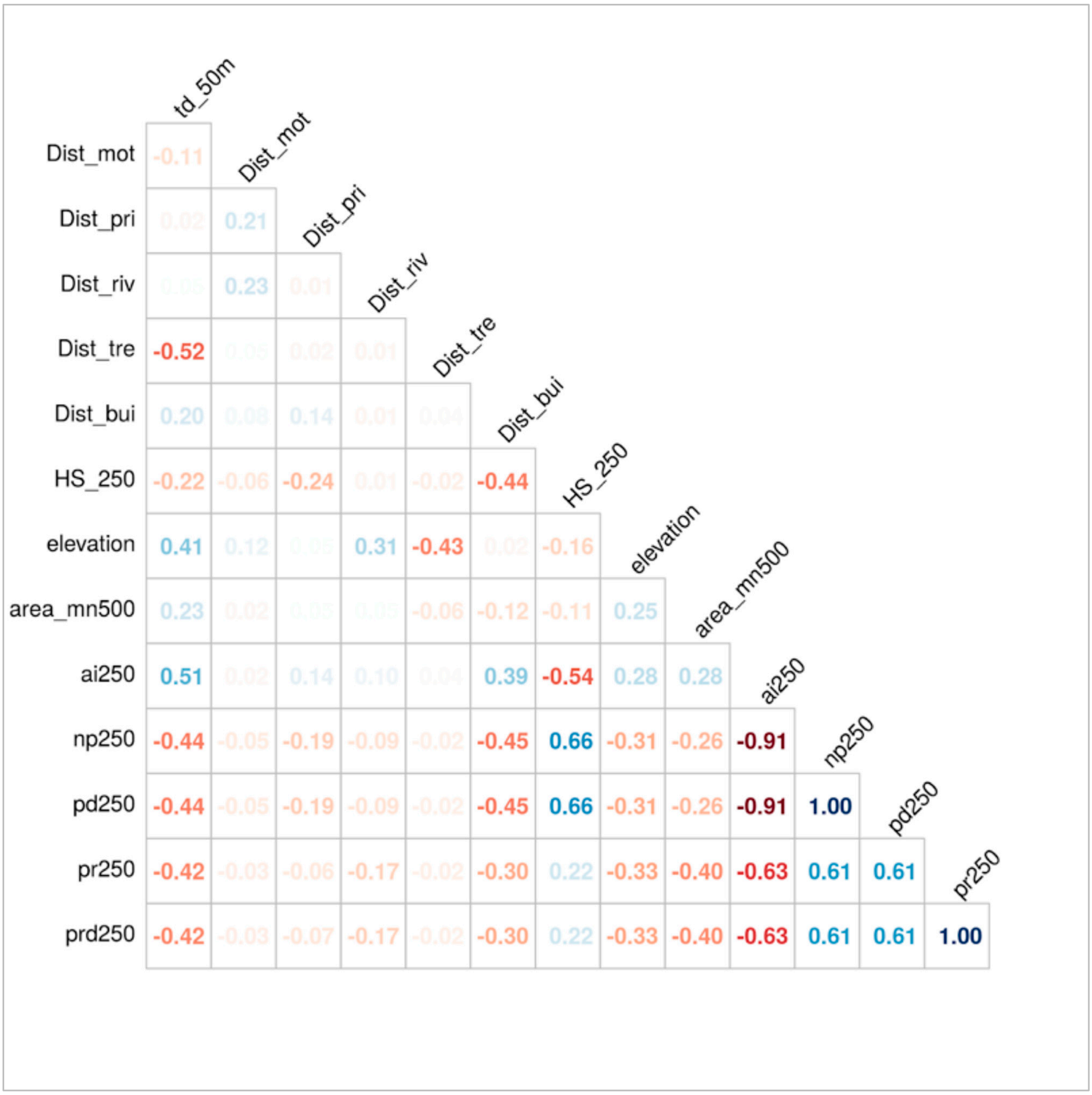

Table S3: Movement patterns.

Identified movement patterns for each of the three GPS-collared wolves in Northern Italy, 2019-2021. The movement patterns were identified inspecting the GPS trajectories in relation to the estimated 95% UD and Net Squared Displacement (NSD) curve.

A - W2357M		
**Period**	**N° days**	**Movement pattern**
17/05/19 – 25/05/19	8	post-release
25/05/19	-	exploration
26/05/19 – 02/06/19	7	settlement
02/06/19 – 08/06/19	6	exploration
08/06/19 – 29/10/19	142	settlement
30/10/19 – 15/11/19	16	pre-dispersal
16/11/19 – 18/11/19	2	settlement
18/11/19 – 10/12/19	21	dispersal

B - W2358F		
**Period**	**N° days**	**Movement pattern**
13/06/19 – 19/06/19	6	post-release
19/06/19 – 22/08/19	64	dispersal
23/08/19 – 20/02/20	181	settlement
21/02/20	-	exploration
21/02/20	-	settlement
22/07/20 – 24/02/20	2	exploration
25/02/20 – 28/02/20	3	settlement
28/02/20 – 01/03/20	2	exploration
01/03/20 – 14/03/20	13	settlement
15/03/20 – 17/03/20	2	exploration
17/03/20 – 28/03/20	11	settlement
29/03/20 – 30/03/20	1	exploration
31/03/20	-	settlement
01/04/20 – 02/04/20	1	exploration
03/04/20 – 23/04/20	20	settlement
23/04/20 – 26/04/20	3	exploration
26/04/20 – 12/06/20	47	settlement

C - W2606		
**Period**	**N° days**	**Movement pattern**
21/04/21 – 06/05/21	15	post-release
07/05/21 – 27/05/21	20	dispersal
28/05/21 – 10/06/21	13	settlement

11/06/21 – 13/06/21	2	exploration
13/06/21 – 20/10/21	129	settlement
21/10/21	-	exploration
21/10/21 – 27/10/21	6	settlement
28/10/21	-	exploration
29/10/21 – 07/11/21	9	settlement
07/11/21	-	exploration
07/11/21 – 09/11/21	2	settlement
09/11/21 – 11/11/21	2	exploration
11/11/21 – 17/11/21	6	settlement
17/11/21	-	exploration
17/11/21 – 23/11/21	6	settlement
23/11/21 – 25/11/21	2	exploration

Figure S3: Minimum daily distance and cumulative line distance.

Minimum daily distance and cumulative line distance, i.e., the sum of the Euclidean distances covered across all successive GPS locations, travelled by three GPS-collared wolves in Northern Italy, 2019-2021.

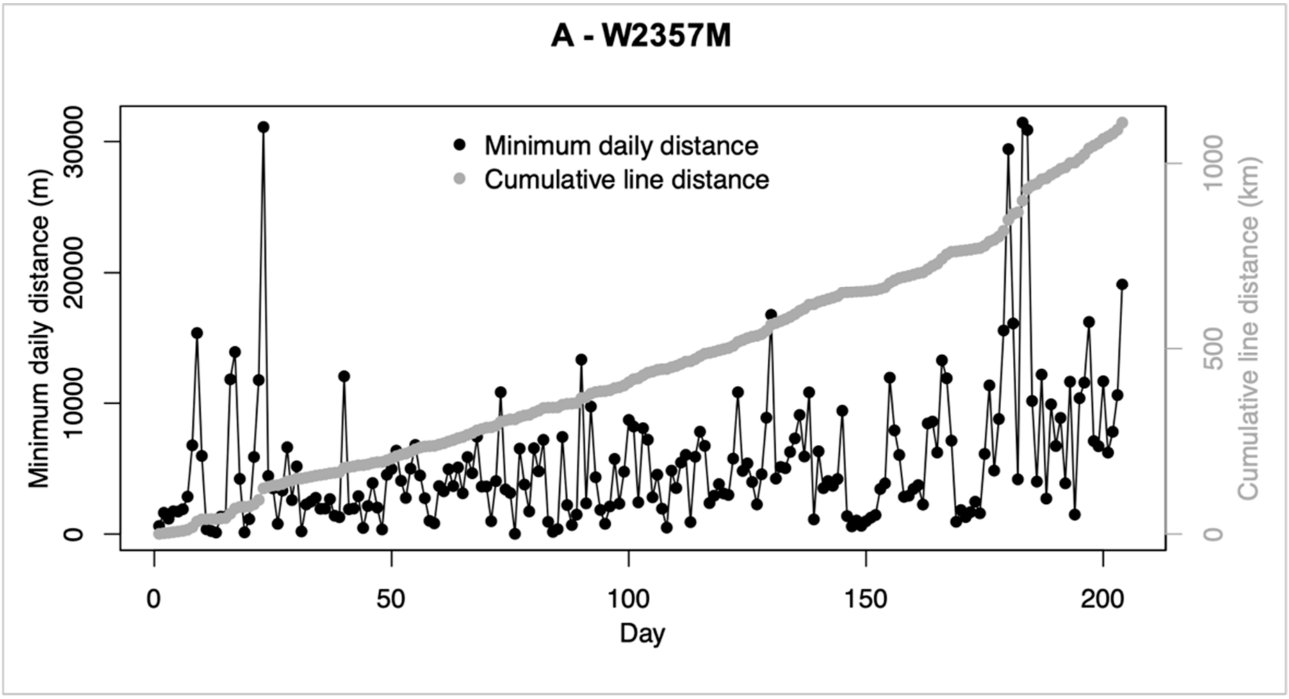

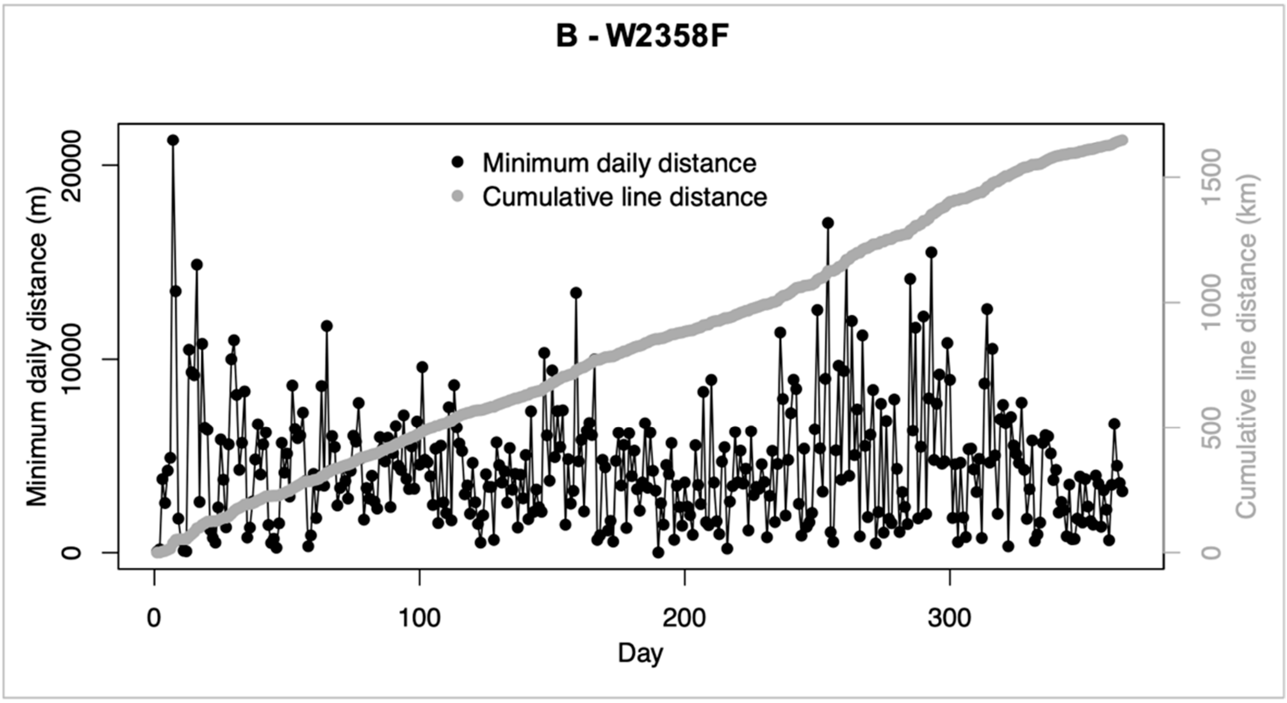

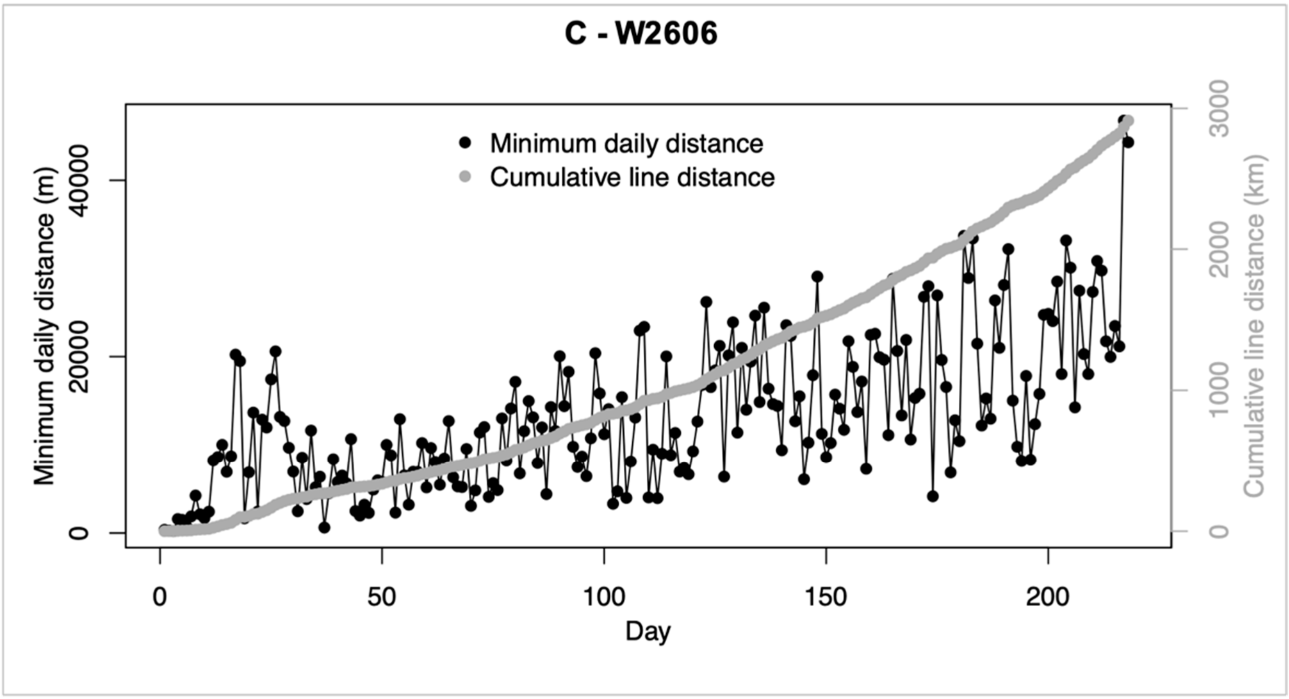
C - W2606
Minimum daily distance
Cumulative line distance
Minimum daily distance (m)
Cumulative line distance (km)
Day
0
20000
40000
0
1000
2000
3000
0
50
100
150
200

Table S4: Relative importance of covariates obtained through multi-model inference.

Model-averaged coefficients (full average) estimated via 'dredge' R function of all fitted generalized linear models with ΔAIC ≤ 2. For each individual, the retained explanatory variables, parameter estimates (with standard error) and significance level are reported. At the bottom, the number of resting sites used to fit the models is reported.

	**Coefficients (Standard Error)**		
	**A - W2357M**	**B - W2358F**	**C - W2606**
Tree Density (50m)	0.924 (0.626)	\	**1.115 (0.411) ****
Human settlement density (250m)	-1.235 (1.197)	\	\
Patch density (250m)	-0.136 (0.369)	0.387 (0.588)	**-1.608 (0.545) ****
Distance from motorways	-0.055 (0.134)	\	16.889 (36.614)
Distance from primary roads	**-0.584 (0.266) ***	\	\
Distance from rivers	\	-0.101 (0.256)	-0.108 (0.211)
Distance from built patch	\	**2.168 (0.737) ****	\
Observations	13	11	16
Note:			*p<0.05; **p<0.01; ***p<0.001
